# Supplementary material for: Systematic Identification of Familial Hypercholesterolaemia in Primary Care—A Systematic Review
Source: J Pers Med. 2021 Apr 15;11(4):302. doi: 10.3390/jpm11040302 (PMC8071332; doi:10.3390/jpm11040302)
Supplement: Supplementary file 1 [file jpm-11-00302-s001.zip › jpm-1135666-supplementary.pdf]

Table S1: Search Strategy

We searched seven electronic sources (PubMed; MEDLINE; Embase; CINAHL; Web of Science; Cystic Fibrosis and Genetic Disorders Group's Inborn Errors of Metabolism Trials Register; Cochrane Central Register of Controlled Trials (CENTRAL) and grey literature sources (ProQuest Dissertations & Theses Global ProQuest; SCOPUS) and Clinical Trials Registries. We searched from inception to March 2020 using a range of subject heading and free text words (see example below for Medline search). Additionally, we hand searched five major journals (*Heart, Atherosclerosis, Journal of Clinical Lipidology, Current Opinion in Lipidology, Journal of Inherited Metabolic Disease*), two online resources from HEART UK 22 and the FH Foundation 23, and relevant guideline developers (National Institute for Health and Care Excellence 24, Scottish Intercollegiate Guidelines Network 25 to identify further eligible studies . Finally, we identified further studies from screening the bibliographies of included studies and any relevant systematic reviews. We also contacted lead authors of the included studies to identify further eligible published or unpublished studies.

Search on Medline (OvidSP)– YEAR 1946-present: 1. (familial or inherited) adj2 hypercholesterol?emia$).tw.
2. Hyperlipoproteinemia Type II/
3. (Hyperlipoprotein?emia$ adj (type II or type IIa or type IIb or type 2 or type 2a or type2b)).tw.
4. 1 or 2 or 3
5. general practice$.tw.
6. GP.tw.
7. (primary adj (health or care)).tw.
8. ((family or community) adj (medicine or practice)).tw.
9. Primary Health Care/
10. exp General Practice/
11. 5 or 6 or 7 or 8 or 9 or 10
12. ((((medical or health or patient$ or electronic) and record$ or database$ or data or audit or reminder$ or tool$)) or (diagnos$ or identif$ or detect$)).tw.
13. 11 and 12
14. laborator$.tw.
15. Laboratories/
16. patholog$.tw.
17. Pathology/or Pathology, Clinical/
18. 14 or 15 or 16 or 17
19. (record$ or database$ or data or audit or tool$ or daignos$ or identif$ or detetct$).tw.
20. 18 and 19
21. screen .tw.
22. mass screening/
23. 21 or 22
24. 23 and (11 or 18)
25. ((family or relative$) and test$).tw.
26. 13 or 20 or 24 or 25
27. 4 and 26

Table S2: Table of Excluded Studies

Study and Year	Title	Design/Setting	Participants	Intervention	Outcomes	Reason for Exclusion (Comparison)
Bell 2012	Opportunistic screening for familial hypercholesterolaemia via a community laboratory	Cross-sectional study<br><br>St John of God Pathology, a private community laboratory in Western Australia	Serum LDL-cholesterol concentrations requested by general practitioners, cardiologists and other specialists	Serum LDL-cholesterol concentrations were reviewed over a one-year period to determine the prevalence of possible FH based on LDL-cholesterol thresholds	FH diagnosis based on MEDPED, Simon-Broome and DLCN cholesterol criteria	No baseline data of usual care
Bell 2013 (Conference Abstract)	Impact of telephoning the requestors of individuals found to be at high risk of familial hypercholesterolaemia	Case – historical control<br><br>St John of God Pathology, a private community laboratory in Western Australia	Individuals with an LDL-cholesterol ≥6.5 mmol/L requested by a GP	A phone call to the requesting GP from the chemical pathologist to highlight their patient's risk of FH and to suggest referral to the Lipid Disorders Clinic	Referral to Lipid Disorder Clinic<br><br>Result of assessment in lipid disorder clinic:<br>-FH diagnosis based on DLCN criteria<br>- genetic testing<br>- lipid lowering therapy	No baseline data of usual care
Bell 2014	Can patients be accurately assessed for familial hypercholesterolaemia in primary care?	Cross-sectional<br><br>Primary care clinics and regional specialist lipid clinic in Western Australia	Individuals at risk of FH were identified by either the laboratory highlighting individuals with elevated LDL-cholesterol, or by using an informatics tool to search general practice databases.	Individuals at risk of FH is examined and data collated by a nurse in primary care, for GP to calculate DLCN score. With lipid specialists independently calculate DLCN score.	Comparison of GPs and lipid specialist diagnosis of FH based on DLCN criteria	No baseline data of usual care
Bell 2015	The potential role of an expert computer system to augment the opportunistic detection of individuals with familial hypercholesterolaemia from a community laboratory	Retrospective analysis<br><br>St John of God Pathology, a private community laboratory in Western Australia	Patients who had lipid profiles requested from the laboratory over 12 months.	An expert computer system was used to search a database consisting of laboratory results and the clinical details provided on the lipid request forms of the current as well as previous requests for each individual.	FH diagnosis based on DLCN:<br>-secondary causes of raised LDL-cholesterol<br>-LDL-cholesterol level<br>-clinical history<br>-family history<br>-statin therapy	No baseline data of usual care

Study and Year	Title	Design/Setting	Participants	Intervention	Outcomes	Reason for Exclusion (Comparison)
Bender 2016	Interpretative comments specifically suggesting specialist referral increase the detection of familial hypercholesterolaemia	This prospective case-control study<br><br>St John of God Pathology (SJGP), Western Australia	Individuals referred by a GP who were found to have an LDL-cholesterol ≥ 6.5 mmol/L	Interpretative comments were added to all the lipid results with the assistance of a computer expert system, with all comments reviewed by chemical pathologists. The cases received an additional recommendation for referral to a Lipid Disorders Clinic, with a subset of cases also had the lipid disorder clinic's fax number included.	Referral to the lipid clinic<br><br>Result of assessment in lipid disorder clinic:<br>-FH diagnosis based on DLCN criteria<br>- genetic testing	No baseline data of usual care
Benn 2012	Familial hypercholesterolaemia in the Danish general population: prevalence, coronary artery disease, and cholesterol-lowering medication	Cross-sectional<br><br><br>The Copenhagen General Population Study, in Denmark.	The study was of an unselected, community-based population.	Data were obtained from a self-completed questionnaire, a brief physical examination, and non-fasting venous blood samples.	FH diagnosis based on DLCN criteria,<br>-genetic testing<br>-LDL-cholesterol levels<br>-risk of CAD<br>-lipid lowering medication	No baseline data of usual care
Casula 2017	Detection of familial hypercholesterolaemia in patients from a general practice database	Cross sectional<br><br>General practice electronic database in Italy.	Individuals with data in general practice electronic health records.	Partial assessment of the DLCN score using available data in the electronic health records. (not include family history and clinical examination)	Diagnosis of FH based on<br>-DLCN criteria<br>-MEDPED criteria	No baseline data of usual care
Elis 2020	The Characteristics of Patients with Possible Familial Hypercholesterolemia-Screening a Large Payer/Provider Healthcare Delivery System	Cross sectional<br><br>Clalit Health Services (CHS), a single largest healthcare fund in Israel	Individuals who are members of Clalit Health Services	Individuals' information in the electronic health records are assessed according to modified General Population MEDPED's total cholesterol level (age-based)	Diagnosis of FH based on modified MEDPED criteria.	No baseline data of usual care

Study and Year	Title	Design/Setting	Participants	Intervention	Outcomes	Reason for Exclusion (Comparison)
Gray 2008	Identifying patients with familial hypercholesterolaemia in primary care: an informatics-based approach in one primary care centre	Cross-sectional<br><br>General Practice in South London, United Kingdom.	Patient registered in a single General Practice	Four computer-based search strategies to identify potential FH cases:<br>- CHD<br>- coded lipid disorder<br>- Statin prescribing<br>- Cholesterol >7 mmol/l<br><br>Selected patients' notes were reviewed by general practitioners and consultant lipidologist	Diagnosis of FH based on DLCN criteria.	No baseline data of usual care
Jayne 2016 (conference abstract)	Specialist familial hypercholesterolaemia (FH) nurses in primary care for identification of FH index cases	Cross-sectional study<br><br>General Practice and Royal Free Hospital, London, UK	3 groups of patients selected.<br>Patients with a Read code of FH; with a total cholesterol >7.5mmol/L or LDL-cholesterol >4.9 mmol/L and triglycerides <3 mmol/L; history of premature cardiovascular disease	Search of Electronic Health Records; selected patients seen in clinic or had telephone consultation	FH diagnosis based on Simon-Broome criteria.<br>Genetic testing of index cases.	No baseline data of usual care
Kirke 2015	Systematic detection of familial hypercholesterolaemia in primary health care: a community based prospective study of three methods	Prospective comparison study<br><br>Primary Health Care settings, Western Australia	Included participants based on intervention:<br>1. patients 18 to 60 years with a total cholesterol >7.5mmol/L or LDL-cholesterol>4.5 mmol/L<br>2. workers at a large local mineral processing operation – selected for further assessment if 2 or more risk factors for coronary artery disease identified<br>3. age 18-70 years, history of cardiac event	Comparison of 3 screening methods:<br>1. pathology laboratory database search;<br>2. workplace health checks<br>3. general practice database search	FH diagnosis based on DLCN criteria	No baseline data of usual care

Study and Year	Title	Design/Setting	Participants	Intervention	Outcomes	Reason for Exclusion (Comparison)
			<60 years, any coronary artery disease, diagnosis of lipid disorder, total cholesterol >7.5 mmol/L, LDL-cholesterol >4.0 mmol/L or prescription for statins			
Qureshi 2016	Feasibility of improving identification of familial hypercholesterolaemia in general practice: intervention development study	Feasibility intervention study<br><br>General practices in central England	Total cholesterol >7.5mmol/L	Educational session in practice; use of opportunistic computer reminders in consultations or universal postal invitation over 6 months to eligible patients invited to complete a family history questionnaire. Those fulfilling the Simon-Broome criteria for possible FH were invited for GP assessment and referred for specialist definitive diagnosis.	FH diagnosis based on Simon-Broome criteria<br>Referral to specialist care, diagnosis of confirmed FH in specialist care;<br>Rates of recruitment of eligible patients, identification of patients with possible FH.	No baseline data of usual care
Safarova 2016	Rapid identification of familial hypercholesterolaemia from electronic health records: The SEARCH study	Cross-sectional study<br><br>Primary Care practices databases, USA	Individuals with LDL-cholesterol >190mg/dL and triglycerides <400mg/dL, without secondary causes of hyperlipidaemia	Search of electronic health records using the SEARCH algorithm combining structured electronic health record data extraction and natural language processing of free text for family history and examination	FH diagnosis based on DLCN criteria	No baseline data of usual care
Shipman 2014 (conference abstract)	Audit of the diagnosis of familial hypercholesterolaemia in primary care	Cross-sectional study<br>General practice West Midlands, UK	Individuals total cholesterol >7.5 mmol/L	Search of electronic health records	In patients identified with raised cholesterol<br>- Disease (Read) code on	No baseline data of usual care

Study and Year	Title	Design/Setting	Participants	Intervention	Outcomes	Reason for Exclusion (Comparison)
					electronic health records<br>- Lifestyle, medication and clinical phenotype	
Troeung 2016	A new electronic screening tool for identifying risk of familial hypercholesterolaemia in general practice	Retrospective study<br><br>Large metropolitan general practice in Western Australia	Active patients (≥ 3 visits within the previous 2 years) and LDL-cholesterol ever tested.	Search of electronic health records using TARB-Ex tool compared with GP manual review. Clinical assessment of high risk patients.	FH diagnosis based on DLCN criteria	No baseline data of usual care
Vickery 2017	Increasing the detection of familial hypercholesterolaemia using general practice electronic databases	Cross-sectional study<br><br>General practices in Western Australia	Patient attended last 2 years; age 18-70 years.	Search of electronic health records using the Canning tool (history of coronary event <60 years old; any coronary arterial disease; lipid disorder; total cholesterol >7.5mmol/L: LDL-cholesterol>4.0 mmol/L; statins prescription)	FH diagnosis based on DLCN criteria	No baseline data of usual care
Zamora 2017	Familial hypercholesterolaemia in a European Mediterranean population - Prevalence and clinical data from 2.5 million primary care patients	Cross-sectional study<br><br>Electronic databases from general practices in Catalonia, Spain.	Individuals aged ≥ 8 years with at least one LDL-cholesterol measurement.	Search of Electronic Health Records for LDL-cholesterol levels	Prevalence of heterozygous and homozygous FH phenotypes based on age-adjusted LDL-cholesterol thresholds FH diagnosis based on MEDPED criteria	No baseline data of usual care

Abbreviations: CAD, coronary artery disease; CHD, coronary heart disease; DLCN, Dutch Lipid Clinic Network criteria; FH, familial hypercholesterolaemia; GP, general practitioner(s); LDL-cholesterol, low-density lipoprotein cholesterol; MEDPED, Make Early Diagnosis to Prevent Early Deaths; SEARCH, Screening Employees And Residents in the Community for Hypercholesterolemia study.

Table S3: Characteristics of included studies

Study and year	Design/ Setting	Participants	Intervention	Outcomes	Comparisons	Main results			Risk of bias (ROBIN S-I)
						Pre-intervention	Post-intervention	Absolute difference (95% CI),*n*	
**Bell et al. 2013**	Uncontrolled before-and-after study<br><br>St. John of God Pathology (SJGP), private not-for-profit organisation providing clinical laboratory services and general practice in Western Australia.	Patients registered in General Practice.<br><br>Gender: Female 68 (70.9%), Male 28 (29.1%)<br><br>*Age (years):* mean ± SD [range]: 53.7 ± 10.7 [26-74]<br><br>Ethnicity: not reported<br><br>*Inclusion criteria*: LDL-Cholesterol concentration≥6.5 mmol/L on a lipid profile requested by a GP.<br><br>*Exclusion criteria*: An identifiable potential secondary cause for the hypercholesterolaemia [hypothyroidism, mixed hyperlipidaemia, nephrotic syndrome and cholestasis) within ±30 days of the LDL-c result	Interpretative comments added to lipid results using expert system and reviewed by a chemical pathologist.	FH diagnosis (Modified DLCN criteria)<br><br>LDL-Cholesterol level<br><br>Referral to specialist	No comments added to lipid results (standard/usual care)	*Definite FH:* 0/96 (0%)	*Definite FH:* 2/96 (2.08%)	*Definite FH:* 2.08% (-2.05 to 7.28%), *n*=96	Low
						Possible FH: 0/96 (0%)	*Possible FH:* 2/96 (2.08%)	*Possible FH:* 2.08% (-2.05 to 7.28%), *n*=96	

Study and year	Design/ Setting	Participants	Intervention	Outcomes	Comparisons	Main results			Risk of bias (ROBIN S-I)
						Pre-intervention	Post-intervention	Absolute difference (95% CI),*n*	
**Green et al. 2016**	Uncontrolled before-and-after study with two sequential interventions.<br><br>NHS Medway CCG comprises 56 General Practices serving in Kent, United Kingdom.	Patients registered in General practice in NHS Medway CCG (approximately 290 000 patients)<br><u>Gender</u>: not reported<br><u>Age</u>: In 2011, 37 200 people were aged >65 years and 4400 aged >85 years<br><u>*Ethnicity*</u>: predominantly white (93.4%, with 2.6% Asian, 1.7% black, 1.4% mixed and 1% Chinese/other).<br><u>*Inclusion criteria*</u>: The FH Audit Tool identified and flagged all patients at potential risk of FH according to elevated cholesterol (total cholesterol > 7.5mmol/L in adults; >4.0mmol/L in children) for further assessment<br><u>*Exclusion criteria*</u>: None stated.	1: computer based reminder message on patient's EHR when cholesterol elevated<br><br>2: FH Nurse Advisor Programme - 2-part process. Part 1 as above; Part 2 involved consultation with nurse to collect further information in order to establish FH diagnosis.	FH diagnosis (Baseline - S-B; Post-intervention – S-B and/or DLCN criteria)	Baseline prevalence of FH determined at study initiation, defined as all patients previously assigned a Read Code for FH according to Simon-Broome criteria.	*EHR search & reminder*			
						Definite FH*: 331/262030 (0.13%)	*Definite FH* 354/199346 (0.18%)	*Definite FH:* 0.05% (0.03 to 0.07%), *n*=262030	Low
						Possible FH:* 12/262030 (0.005%)	*Possible FH:* 88/199346 (0.04%)	*Possible FH:* 0.04% (-0.03 to 0.05%), *n*=262030	
						EHR search & reminder + nurse intervention			
						Definite FH:* 331/262030 (0.13%)	*Definite FH:* 546/281655 (0.19%)	*Definite FH:* 0.07% (0.05 to 0.09%) *n*=262030	Moderate
						Possible FH:* 12/262030 (0.005%)<br><br>**S-B criteria does not include Probable FH*	*Possible FH:* 147/281655 (0.05%)	*Possible FH:* 0.05% (0.04 to 0.06%), *n*=262030	
							Probable FH: 83/281655 (0.03%)	n/a	

Study and year	Design/ Setting	Participants	Intervention	Outcomes	Comparisons	Main results			Risk of bias (ROBINS-I)
						Pre-intervention	Post-intervention	Absolute difference (95% CI),*n*	
**Weng et al. 2018**	Uncontrolled before-and-after study<br><br>Six General Practices (four inner city, one suburban, one rural) in Nottingham, East Midlands, United Kingdom	Patients registered in 6 General practices.<br><br>831 identified, 118 patients medical records accessed<br><br>__Gender__: Number (%) of male and female: 46 (39%) Male and 72 (61%)<br><br>*__Age (years) Average (SD):__* male 58 (9.0), female 56 (7.5)<br><br>__Ethnicity__: not reported<br><br>*__Inclusion criteria__*: Patients aged ≥18 years, with a total cholesterol level ≥7.5 mmol/L documented in their primary care electronic health records (EHR).<br><br>*__Exclusion criteria:__* Patients with a confirmed diagnosis of FH	*Combined approach:* Opportunistic recruitment following computer-based reminder message that appears when GPs accessed eligible participants' records in patient consultations. Systematic postal recruitment of eligible patients two months after start of intervention.	FH diagnosis (S-B criteria)<br><br>*Cholesterol levels*<br>-Mean total cholesterol in mmol/L (SD),<br>-Mean LDL cholesterol in mmol/L (SD).<br><br>*Management*<br>-No. prescribed any statins;<br>-No. prescribed high potency statins;	Same 118 participants with Cholesterol ≥7.5 mmol/L after the release of the NICE FH guidelines in November 2008 ('before' pre-intervention phase)	*Definite FH:* 0/118 (0%)	*Definite FH:* 2/118 (1.69%)	*Definite FH:* 1.69% (-1.69 to 5.97%), *n*=118	Low
						Possible FH: 0/118 (0%)	*Possible FH:* 30/118 (25.42%)	*Possible FH:* 25.42% (17.75 to 33.97%), *n*=118	
